# Supplementary material for: Patterns of Intron Gain and Loss in Fungi
Source: PLoS Biol. 2004 Nov 30;2(12):e422. doi: 10.1371/journal.pbio.0020422 (PMC532390; doi:10.1371/journal.pbio.0020422)
Supplement: Table S1 — Also available at http://genes.mit.edu/NielsenEtAl/. (4.3 MB ZIP). [file pbio.0020422.st001.zip › NielsenEtAl/html/colors.html]

## Classifications and colors of intron positons


### GAINED INTRON

This intron position is a raw intron gain event because it appears in in only one organism, and that organism is not the outgroup. 

---


### POSITION FAILED FILTER: LOW CONSERVATION

This intron position failed the filter because it is not in a conserved region of the protein. Intron positions are only accepted if there is 30% identity and 50% similarity in the 10 residues both upstream and downstream. This also includes a small number of introns that failed because they were adjacent to gaps in the amino acid alignment.

---


### OTHER INTRON

This intron passed the filter but was not included in any of the raw counts and was not totally conserved.

---


### TOTALLY CONSERVED INTRON

This intron position is conserved among all the organisms.

---


### POSITION FAILED FILTER: NEARBY INTRON

This intron position failed because there is another intron within a 10-residue window and without conservation inbetween. Positions with nearby introns are accepted only if there is at least 30% identity and 50% similarity between them, if they are not adjacent to any gaps in the amino acid alignment, and if they are separated by at at least two residues. 

---


### LOST INTRON

This intron position is a raw intron loss event because it appears in all but one organism, and that organism is not the outgroup. 

---
